# High sensitivity glycomics in biomedicine

**DOI:** 10.1002/mas.21730

**Published:** 2021-09-07

**Authors:** Guinevere S. M. Lageveen‐Kammeijer, Bernhard Kuster, Dietmar Reusch, Manfred Wuhrer

**Affiliations:** ^1^ Leiden University Medical Center Center for Proteomics and Metabolomics Leiden The Netherlands; ^2^ Chair for Proteomics and Bioanalytics Technical University of Munich Freising Germany; ^3^ Pharma Technical Development Europe Roche Diagnostics GmbH Penzberg Germany

**Keywords:** glycomics, high sensitivity, mass spectrometry

## Abstract

Many analytical challenges in biomedicine arise from the generally high heterogeneity and complexity of glycan‐ and glycoconjugate‐containing samples, which are often only available in minute amounts. Therefore, highly sensitive workflows and detection methods are required. In this review mass spectrometric workflows and detection methods are evaluated for glycans and glycoproteins. Furthermore, glycomic methodologies and innovations that are tailored for enzymatic treatments, chemical derivatization, purification, separation, and detection at high sensitivity are highlighted. The discussion is focused on the analysis of mammalian *N*‐linked and GalNAc‐type *O*‐linked glycans.

**Abbreviations**
2‐AA: 2‐ aminobenzoic acid
2‐AB: 2‐aminobenzoamide
APTS: 9‐aminopyrene‐1,4,6‐trisulfonic acid
CE: capillary electrophoresis
DEN: dopant‐enriched nitrogen
ESI: electrospray ionization
FASP: filter‐based sample preparation methods
FFPE: formalin‐fixed and Paraffin‐embedded
FLD: fluorescence detection
GirP: Girard's reagent P
HILIC: hydrophilic interaction liquid chromatography
IgG: immunoglobulin G
IMS: ion mobility spectrometry
LC: liquid chromatography
LIF: laser induced fluorescence
MALDI: matrix‐assisted laser desorption/ionization
MS: mass spectrometry
MSI: mass spectrometry imaging
PCMF: post‐column make‐up flow
PD: pharmacodynamic
PGC: porous graphitized carbon
PK: pharmacokinetic
PSA: prostate‐specific antigen
PVDF: polyvinylidene fluoride
RFMS: RapiFluor‐MS
RP: reversed phase
SEC: size exclusion chromatography
SPE: solid phase extraction
TOF: time‐of‐flight
UPLC: ultra‐performance liquid chromatography

## 1 INTRODUCTION

### 1.1 The need for high sensitivity glycomics

**Figure 1:**
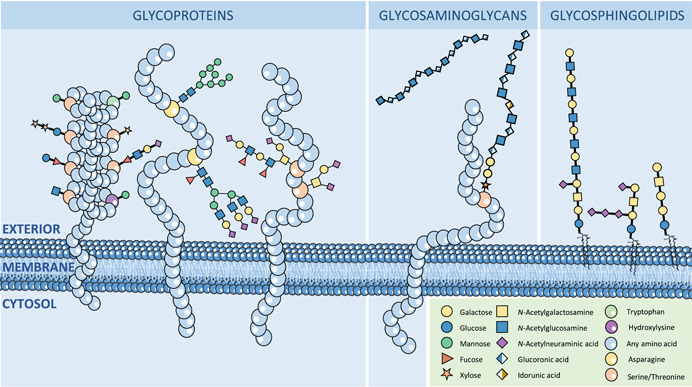
Major types of glycosylation on human cell membranes [Color figure can be viewed at wileyonlinelibrary.com]

Glycoconjugates play a major role in a diverse range of complex biological processes including immune recognition of pathogens, cell–matrix and cell–cell interactions, cellular differentiation, and proliferation (Varki, 2017). Protein‐linked glycans which mainly occur as *N*‐linked and mucin‐type *O*‐linked glycans (Figure 1) are involved in directing intracellular protein trafficking and targeting, modulating protein‐protein interactions, and protein clearance. Next to the GalNAc‐linked *O*‐glycans, also other protein linked *O*‐glycans exist such as *O*‐fucose, *O*‐glucose, *O‐N‐*acetylglucosamine, *O*‐mannose, and *O*‐xylose (Figure 1) (Schjoldager et al., 2020). Moreover, additional glycan classes such as lipid‐linked glycans and glycosaminoglycans (Figure 1) are likewise involved in a range of biological processes such as inflammation, molecular signaling, cell proliferation, and tissue architecture (Puri et al., 2020; Zhang et al. 2019). The focus of this review will be on the most abundant mammalian glycan classes (*N*‐ and *O*‐GalNAc linked glycans).

In accordance with their multiple biological functions, glycans are implicated in most human diseases including cancer, autoimmune as well as cardiovascular and metabolic disorders (Reily et al., 2019; Rudman et al., 2019; Walt et al., 2012). Regarding the role of glycans in cancer, cellular transformation, and the development of cancer are associated with changes in the glycosylation, contributing to various disease processes including proliferation and metastasis (Pinho & Reis, 2015; Zhang, Ten Dijke, et al. 2020). Interestingly, most cancer biomarkers that are routinely detected in blood for diagnosis and prognosis are glycoproteins (e.g., prostate‐specific antigen for the early detection of prostate cancer), the same accounts for biomarkers used to monitor therapy treatment (e.g., carcinoembryonic antigen for various types of cancer) (Hanna‐Sawires et al., 2021; Kirwan et al., 2015).

In the field of biomedicine and biopharma, glycan analysis is relevant for many different purposes. Namely, most biopharmaceuticals are glycoproteins and often the glycan part has an impact on the biological activity. Consequently, characterization of the glycosylation of biopharmaceuticals is mandatory as glycans are considered to be critical quality attributes (Reusch & Tejada, 2015). For example, the absence of fucose in the *N*‐glycans of a therapeutic antibody may lead to a tremendous increase in antibody dependent cell‐mediated cytotoxicity (Chung et al., 2012). The availability of high‐throughput methods is, therefore, of high importance for clone selection, process development and lot release. Moreover, glycans on therapeutic glycoproteins often have an impact on safety, immunogenicity and pharmacokinetic/pharmacodynamic (PK/PD) behavior. Recently, there is an increasing interest in proteoform‐resolved PK analysis of biopharmaceuticals to assess the specific fates of different glycoforms. These analyses often come with formidable challenges due to the complexity of the biological matrices and the target molecules. In addition, often very limited amounts of material are available (Falck et al., 2021; Han et al., 2021).

On a genomic and transcriptomic level, various molecular analyses can be performed on large numbers of samples at ultrahigh sensitivity, for example at the single cell level or at the level of small cell populations (Aldridge & Teichmann, 2020). While proteomic and metabolomic analyses are lagging behind regarding throughput, sensitivity levels are being reached that enable the analysis of small cell populations down to single cells, providing valuable qualitative and quantitative information (Duncan et al., 2019; Fessenden, 2016; Slavov, 2021; Williams et al., 2020).

On the contrary, these sensitivity levels are only rarely reached for molecular glycomic analyses, such as in a proof‐of‐concept study that demonstrated single molecule glycan detection, visualization, and differentiation upon using mass spectrometry (MS) combined with electron microscopy detection for sample preparation, ionization and vacuum separation (Wu et al., 2020). Also, capillary electrophoresis laser‐induced fluorescence detection (CE‐LIF) of reducing‐end labeled glycans using highly fluorescent dyes has shown sensitivities compatible with single‐cell analysis (Whitmore et al., 2007). However, these high sensitivity approaches have not yet found their way into single molecule or single cell applications, in part due to the lack of throughput and suitable sample preparation methods.

Instead, glycomic signatures are often inferred from the transcriptomic analysis of “glycogenes” such as for the glycosyltransferases (Dusoswa et al., 2020; Nairn et al., 2008). In addition, cellular and tissue‐level glycomic signatures are often determined using glycan‐binding proteins in, for example, immunohistochemistry (Jeschke et al., 2005; Lenos et al., 2015), solid‐phase proximity ligation experiments (de Oliveira et al., 2018), or flow cytometry (Zhang, van Die, et al. 2020). These approaches, however, often do not provide detailed structural features of the glycan, implicating the need for high sensitivity and higher resolution glycomic methodologies that allow a comprehensive assessment of glycomic signatures.

**Figure 2:**
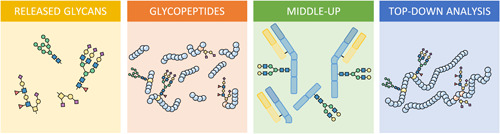
Various analyte classes that can be studied in glycomic research [Color figure can be viewed at wileyonlinelibrary.com]

In this review recent developments in the field of high sensitivity based glycomics are highlighted. High sensitivity MS glycomics builds on various innovations of the past few decades, including in‐gel enzymatic *N*‐glycan release followed by MS for the characterization of the *N*‐glycosylation of proteins as pioneered by Harvey and coworkers (Kuster et al., 2001; Wheeler & Harvey, 2001). Other important innovations involve simple sample preparation methods such as enzymatic *N*‐glycan release in combination with filter‐based micro dialysis desalting steps for facile characterization of *N*‐glycans by matrix‐assisted laser desorption/ionization (MALDI)‐MS or direct infusion electrospray ionization (ESI)‐MS (Wheeler et al., 2009). Here, we will provide a perspective on high sensitivity glycomics covering the analysis of not only released glycans but also glycopeptide‐based glycoproteomics and intact glycoprotein analysis by MS (Figure 2).

## 2 NON‐MS BASED APPROACHES

Flow cytometry, using glycan‐binding proteins such as plant lectins and monoclonal antibodies, is often applied for non‐MS‐based glycosylation analysis of cells. In cases where, for example, human lectins are used, the data may have direct implications for cellular interactions relying on these glycans, making these types of studies highly relevant in the context of, for example, microbial and cellular glycoimmunology and cancer glycobiology (Holst et al., 2017). Similarly, lectin arrays—be it slide‐based or bead‐based—are applied to type the glycosylation of glycoproteins in biopharma (Roucka et al., 2016) and biomedicine (Jegouzo et al., 2020; Pilobello & Mahal, 2007).

To deduce the glycomic makeup of a cell, the cellular glycosylation can be characterized using transcriptomic signatures (Bennun et al., 2013). However, protein glycosylation is a complex and multifactorial process which is not only influenced by enzyme activity but also by substrate availability as well as the competition between them (Varki, 2017). Moreover, the regulatory mechanisms are still poorly understood, even for the best‐studied model proteins including immunoglobulin G (IgG) (Klarić et al., 2020).

The gold standard method for glycan fluorescence detection is hydrophilic interaction liquid chromatography (HILIC) of 2‐aminobenzamide (2‐AB)‐labeled glycans which exhibits limited sensitivity (typical limits of detection are low femtomole quantities of glycans on column; Table 1) (Wuhrer et al., 2004). In contrast, CE‐LIF detection is a highly sensitive method for the detection of labeled glycans reaching down to yoctomole levels (Table 1) (Whitmore et al., 2007), albeit this was not embedded in an analytical workflow. Previously, sensitivities have been reported in the low attomole range (Guttman, 1996), relying on the 9‐aminopyrene‐1,4,6‐trisulfonic acid (APTS) tag (Table 1) or related tags with multiple negative charges. However, it should be noted that, while CE is able to provide high sensitivity, the sensitivity of the overall workflow is often confined by the limited sample amount being injected and consumed for the analysis. The sample volume that is needed to perform the analysis is generally in the range of a few microliters of which only low nanoliter volumes are consumed (Lageveen‐Kammeijer et al., 2019). This often results in the consumption of <2% of the total sample volume, this could be considered as a disadvantage of the platform. On the other hand, samples can be reanalyzed on the same platform or even by different platforms.

**Table 1:** Overview of glycome profiling tools

Glycome profiling tool	Analytes	Sensitivity	Advantages	Disadvantages	Reference
Released glycans					
Non‐MS based					
CE‐LIF	Glycosphingolipid	Mid ymol	Ultrahigh sensitivity High throughput Short analysis time High dynamic range Isomeric separation Small sample volume	Indirect structural information Labeling necessary Small sample volume requires sensitive detection	Whitmore et al. (2007)
CGE‐LIF	APTS‐labeled	Low amol	High sensitivity Separation of isomeric species	High sample volume (~3 µl) needed	Guttman (1996)
DNA sequencer	APTS‐labeled	Low fmol	High sensitivity Isomeric separation Short analysis time		Callewaert et al. (2001)
HILIC‐UPLC‐FLD	2‐AB	Low fmol	High dynamic range Isomeric separation Database availability	Low to medium throughput Labeling advantageous	Wuhrer et al. (2004); Royle et al. (2002); Rudd and Dwek (1997)
MS‐based					
CE‐MS—DEN gas	Negative mode (APTS labeled)	Not defined	High sensitivity Isomeric separation	High volume (~3 µl) needed Low throughput Labeling needed	Marie et al. (2021)
	Positive mode (glycan standard; GirP labeled)	Low amol			Lageveen‐Kammeijer et al. (2019)
nanoRPLC‐MS	Positive mode	Mid amol	Short analysis time	Medium throughput	Unpublished
nanoLC‐PGC‐MS—DEN gas	Negative mode (glycan standard)	Mid fmol	Isomeric separation Diagnostic ions	Extensive sample preparation Low throughput Labeling needed	Madunic et al. (2021)
MALDI‐TOF‐MS	Positive mode (glycan standard)	Mid fmol	Small sample volume Distinction of sialic acid isomers (derivatization) High throughput		Lageveen‐Kammeijer et al. (2019)
MALDI‐MSI	Negative mode (glycan standard)	Low fmol	Diagnostic ions (neg mode)	Low throughput	Heijs, Potthoff, et al. (2020)
	Positive mode (glycan standard)	Mid amol			Heijs, Potthoff, et al. (2020)
MALDI‐2 (MSI)	Negative mode (glycan standard)	Low amol	Diagnostic ions (neg mode)	Low throughput	Heijs, Potthoff, et al. (2020)
Glycopeptide analysis					
MS based					
CE‐MS—DEN gas	Positive mode (IgG glycopeptides)	Low amol	Distinction of sialic acid isomers	High volume (~3 µl) needed Low throughput	Kammeijer et al. (2016)
nanoLC‐MS	Positive mode (IgG glycopeptides)	Low fmol		Low throughput	Kammeijer et al. (2016)

A variety of different hardware setups exist, including DNA analyzer platforms used for glycan analysis and chip‐based solutions (Ruhaak et al., 2010; Sarkozy et al., 2020). Providing high‐throughput applications, either due to multiplexing (e.g., DNA analyzer platforms) (Ruhaak et al., 2010) or due to very short run times of only several minutes (Zhuang et al. 2007). CE‐LIF can achieve similar precision (Reiding et al., 2019) as HILIC with fluorescence detection (FLD), making it a very attractive method for non‐MS‐based high sensitivity glycomics.

## 3 MS GLYCOMICS: SAMPLE PREPARATION

Next to the choice of a suitable detection method, the choice of an appropriate, sensitive sample preparation method is key for achieving high sensitivity workflows. Characteristics of such sample preparation workflows are miniaturization and the minimization of losses by diminishing the number of (sample handling) steps. Also, to achieve ultimate sensitivity of the overall workflow, it is desirable that a large portion of the prepared sample can be used for detection/analysis. Miniaturization generally also facilitates multiplexing (e.g., sample preparation in a 96‐ or 384‐well plates) and tends to speed up and simplify the sample preparation procedures. For example, tedious vacuum centrifugation steps can be replaced by a simple evaporation step (Váradi et al., 2014). In the following section we will discuss several key aspects of high sensitivity glycomics sample preparation methods, with a focus on glycomic methods and their corresponding clean‐up steps.

### 3.1 Glycoprotein immobilization

Immobilization of glycoproteins is an important aspect of many *N*‐ and *O*‐glycan sample preparation methods. This includes covalent immobilization of glycoproteins on beads (Yang et al., 2017) and, the more widely used procedure, immobilization of proteins on polyvinylidene fluoride (PVDF) membranes (Jensen et al., 2012). Both approaches are scalable by adjusting the number of beads and the size of the membrane piece. Immobilization on the PVDF membranes may be in part confounded by the presence of detergents in the sample, as the detergent coats the membrane and prevents adsorption of the protein, reducing its immobilization. Especially in the case of minute amounts, the ionic detergent decreases the protein binding (e.g., sodium dodecyl sulphate [SDS]; unpublished data). Immobilization on beads is often achieved using amine‐targeting chemistry and buffers, containing ammonia or primary amines which interfere with the immobilization. Glycoprotein immobilization allows solid‐phase glycan derivatization with facile cleanup (Yang et al., 2017) and enzymatic *N*‐glycan release, eventually followed by *O*‐glycan release applying reductive beta‐elimination (Jensen et al., 2012).

### 3.2 Glycan release

To study the glycosylation of biospecimens, various techniques are available that either chemically or enzymatically release the glycans from their conjugates. Before the release, the tremendous glycan heterogeneity of a conjugate should be considered, including its macro‐ (site occupancy) and microheterogeneity (variation of glycans at a specific site). For example, it should be investigated whether the release can be performed in a nonselective manner, introducing no alterations of the glycan moiety, and providing a representative profile of the conjugate. Often an enzymatic approach is selected, that enables the release of *N*‐glycans in‐solution, with PNGase F being the most commonly used enzyme for mammalian samples. This enzyme exhibits a wide specificity, yet an α1,3‐linked core fucose will not be accepted, which is commonly found in, for example, plants and insects. In general, only 5 up to 10 microliter of serum/plasma as starting material is required (approximately 70  microgram of protein content per microliter) to obtain a representative glycomic profile for most MS platforms (Adua et al., 2021; Reiding et al., 2019; Vreeker et al., 2018).

While a wide repertoire of additional enzymes is available for the release of *N*‐glycans, the possibilities of releasing *O*‐linked glycans remain limited (Karlsson et al., 2017; Mulagapati et al., 2017; Wilkinson & Saldova, 2020). While several enzymes are now commercially available, they often show a restricted specificity (e.g., only core 1 and 3 *O*‐glycans) limiting their applicability. Instead, a chemical release is often selected, such as hydrazinolysis or beta‐elimination, but these procedures generally degrade the protein, may affect glycan integrity, require hazardous chemicals and often involve extensive clean‐up steps (Merry & Astrautsova, 2003; Wilkinson & Saldova, 2020).

Hinneburg et al. (2017) presented a highly sensitive method to simultaneously release *N*‐ and *O*‐glycans from a tissue section. In addition, they demonstrated that only 2,000 cells from formalin‐fixed and paraffin‐embedded (FFPE) sections were required to obtain reliable and quantitative glycomic profiles using laser capture microdissection (Hinneburg et al., 2017). With this workflow distinct glycan expressions were found between tumor (hepatocellular carcinoma) and nontumor (noncancer hepatic tissue) tissues and revealed that similar glycomic profiles could be obtained for unstained versus hematoxylin and eosin stained FFPE tissues. However, slight differences were noted for the glycomic profiles when fresh frozen tissues were used. Another protocol, published by the Lebrilla lab (Li et al., 2020), demonstrated that, next to the sequential release of *N*‐ and *O*‐glycans, also the glycan head groups of glycosphingolipids can be characterized by using the same enriched membrane fraction. To allow a comprehensive analysis of all these analyte classes by liquid chromatography (LC)‐MS, the workflow requires around one million cells as starting material. It should be noted that before cell membrane extraction, lysis of the cells and tissues is required.

### 3.3 In‐gel *N*‐glycan release

In cases where limited sample amounts are available, or when further purification is desired, *N*‐glycan release could be performed by using an in‐gel *N*‐glycan release. This method was pioneered by Kuster et al. (1997) building on the success of in‐gel proteomics sample preparation. An important breakthrough paving the way for the success of MS‐based proteomics was the establishment of in‐gel protease digestion as a sample preparation technique (Shevchenko et al., 1996; Wilm et al., 1996). This was a key step because it brought together a workhorse protein separation technique in biology (SDS gel electrophoresis) and MS (a sensitive and multianalyte detection system). The idea was soon thereafter extended to release *N*‐glycans from gel‐separated proteins using PNGase F (Kuster et al., 1997, 1998; Wheeler & Harvey, 2001) which improved sample preparation along several lines at once. Glycan analysis is generally performed on a pure protein to reveal protein‐specific glycosylation profiles. Given that most glycoproteins are integral plasma membrane proteins, the use of detergents is often mandatory for efficient extraction. Detergents are, however, generally incompatible with MS detection. In addition, membrane protein purification by chromatographic or other methods remains difficult which often means that a particular glycoprotein of interest cannot be purified to homogeneity. The use of sodium dodecyl sulphate polyacrylamide gel electrophoresis (SDS‐PAGE) as a sample preparation step solves both issues at the same time. Gel electrophoresis tolerates samples of practically any buffer composition and provides an additional, standardized step of protein purification. Gels are also safe containers for proteins, which allow the efficient removal of any buffer component that may interfere with downstream sample derivatization or analytical techniques. In‐gel *N*‐glycan release also improved the overall sensitivity of the analytical workflow as quantities in the range of 1 up to 100  microgram protein were sufficient to obtain *N*‐glycan profiles (Clark et al., 1999; Kuster et al., 2001, 2000; Rudd et al., 1999). Further advantageous features of the in‐gel deglycosylation method include the fact that protein identification by standard proteomic approaches can still be performed efficiently, including *N*‐glycan site localization when considering the Asn‐Asp conversion that comes with PNGase F digestion. The latter may be further aided by releasing the glycans in the presence of 50% ^18^O‐labeled water to leave a characteristic isotopic signature that can be recognized by MS (Kuster & Mann, 1999; Liu et al., 2010). Even if the focus of the analysis is the protein and not the glycans, in‐gel deglycosylation can be a powerful step as it removes the often‐large glycan “shield” that could render tryptic cleavage sites due to inaccessibility to the protease. One of the downsides of the approach is that it does not reveal site‐specific *N*‐glycan patterns. Moreover, the assumption that the band is “clean” and only contains a single protein may often not be justified, as proteins with similar properties would comigrate. If in‐gel deglycosylation is performed on such a band, a total glycan profile would be obtained, and it cannot be distinguished whether a specific glycan is corresponding to the protein of interest or another comigrating protein. However, this may be mitigated by a proteomic analysis of the same sample that estimates how much of the total protein in a particular gel band is represented by the protein of interest. SDS‐PAGE is also difficult to scale‐up which is mostly a minor issue given the continuous thrive for working with rare biological material. These shortcomings aside, the method has been adopted rapidly by the field (Duarte et al., 2021; Royle et al., 2003; Rudd et al., 1999). Because ultra‐performance liquid chromatography (UPLC)‐FLD, MALDI‐time‐of‐flight (TOF)‐MS and nanoESI‐TOF‐MS/MS show considerable orthogonality, their integration for *N*‐glycosylation analysis of gel‐separated proteins proved highly valuable (Ritchie et al., 2010). In‐gel *N*‐glycan release may be combined with sialic acid derivatization and MALDI‐TOF‐MS analysis after sialic acid stabilization (von der Ohe et al., 2002). With the use of specific sialic acid derivatization workflows, sialic acid linkage differentiation can be achieved (Bieberich, 2014; Marti Fernandez et al., 2019; Stavenhagen et al., 2018). The approach is versatile and has been applied to Coomassie‐stained protein bands in SDS‐PAGE using fluorescent labeling and HILIC‐HPLC‐FLD or CE‐LIF, optionally combined with an array of exoglycosidase treatments for glycan sequencing (Aghamohseni et al., 2014; Royle et al., 2007; Schwarzer et al., 2008). In‐gel *N*‐glycan release has also been applied to 2D‐gel electrophoresis revealing differential glycosylation of charge‐resolved haptoglobin proteoforms (He et al., 2006). Another valuable addition is negative‐mode nanoESI‐TOF‐MS/MS as the tandem mass spectra often allow the dissection of *N*‐glycan structures (Chandler et al., 2014; Harvey et al., 2008). Recently, in‐gel enzymatic *N*‐glycan release has successfully been combined with a high‐sensitivity glycoanalytical workflow including linkage‐specific sialic acid derivatization, Girard's reagent P labeling and capillary electrophoresis mass spectrometry (CE‐MS; Duarte et al., 2021).

While in‐gel *N*‐glycan release has its place, it should be noted that in‐gel trypsin digestion followed by bottom‐up glycoproteomic workflows is gaining importance in the glycosylation analysis of gel‐separated proteins (Duarte et al., 2021; Rodrigues et al., 2021). Yet, released *N*‐glycan analysis has a role as it often allows a more thorough analysis of the glycan structure as compared to glycopeptide analysis, in particular when powerful glycan separation and tandem MS approaches such as porous graphitized carbon (PGC)‐LC‐MS/MS are applied (Stadlmann et al., 2008; Wuhrer, 2013). As an alternative, in‐gel hydrazinolysis may be applied for chemical release of *N*‐glycans (He et al., 2006), followed by fluorescent labeling and HPLC‐FLD. The approach features, surprisingly, good sensitivity with high‐quality glycosylation profiles achieved with as little as one  microgram of human IgG. Due to the suitability of hydrazinolysis for the release of *O*‐glycans next to *N*‐glycans, in‐gel hydrazinolysis has potential for a more comprehensive glycosylation profiling of gel‐separated proteins at the released glycan level.

Glycomic analysis of gel‐separated proteins has alternatively been demonstrated after electroblotting onto a PVDF membrane via spatially resolved *N*‐glycan release and on‐membrane MALDI‐TOF‐MS (Kimura et al., 2007). Interestingly, the approach allowed multiplexing by applying a range of different enzymes in parallel, including PNGase F and trypsin, supporting parallel glycomic and proteomic characterization of individual protein spots in 2D‐gel electrophoresis. While the approach has not been followed up a lot, it may have potential when integrated with recent glycomic MS imaging (MSI) workflows (see Section 4.5). Wilson et al. (2002) similarly used 2D‐gel electrophoresis with electroblotting for glycomics analysis, applying sequentially PNGase F treatment and reductive beta‐elimination for *N*‐ and *O*‐glycan release, followed by PGC‐LC‐MS/MS, achieving the charge isoform‐resolved, comprehensive glycomic characterization of various major human plasma glycoproteins.

Filter‐based sample preparation methods which are widely used in proteomics (Wiśniewski et al., 2009) are also are amenable for high sensitivity *N*‐glycomics analyses (Abdul Rahman et al., 2014; Feng et al., 2019; Hecht et al., 2015) as well as the analysis of proteoglycans applying, for example, chondroitinase ABC (Sethi et al., 2020).

### 3.4 Labeling procedures

**Figure 3:**
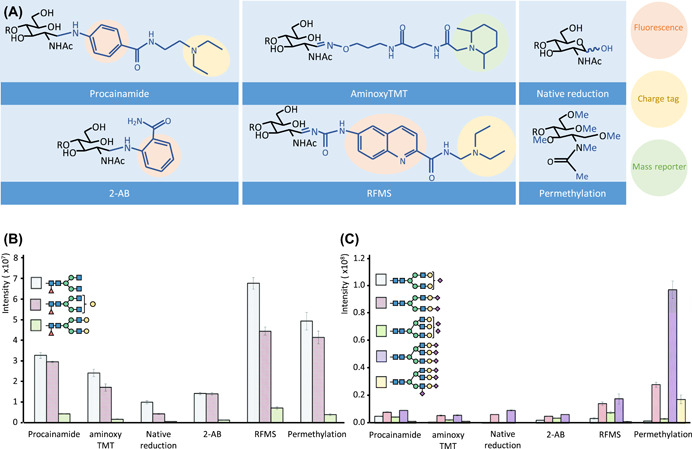
Overview of selected glycan derivatization approaches. (A) representation of different derivatization products; R, 4‐linked saccharide. Bar graphs illustrate the observed intensities of differently derivatized glycans released from (B) IgG and (C) fetuin analyzed by LC‐MS. Adapted from (Zhou et al. 2017) with permission from The Royal Society of Chemistry. LC‐MS, liquid chromatography mass spectrometry [Color figure can be viewed at wileyonlinelibrary.com]

To facilitate glycan detection after the usage of separation platforms such as CE or LC, often a chromophore or fluorophore is introduced. Specific derivatization strategies may also help to improve sensitivity in MS detection, such as the addition of a charged group at the reducing end or by increasing the hydrophobicity and stabilizing the sialic acid residues through permethylation. Moreover, dependent on the labeling type, this may facilitate structural elucidation by improving fragmentation of the analyte. Zhou et al. (2017) recently compared the analysis of released glycans after reduction, permethylation, and the application of various reducing‐end tags (Figure 3). A gain in intensity is observed when a tag is added to the reducing end and when the hydroxyl groups are derivatized compared to the MS analysis of reduced *N*‐glycans. However, sample loss might occur due to incomplete reactions that are associated with the labeling procedure, especially for the low abundant glycans. Overall, the usage of charged tags (*Rapi*Fluor‐MS; RFMS (Lauber et al., 2015) or procainamide) provided the highest sensitivity for nonsialylated glycan analysis (Figure 3B). While permethylation was found to outperform these approaches when sialylated species are of interest (Figure 3C), indicating the importance of stabilization of sialic acid and removal of the associated negative charge.

Permethylated glycans show very favorable ionization properties in ESI and MALDI, like other relatively hydrophobic analytes. In ESI, both proton and sodium adducts of permethylated glycans are readily formed (Ashline et al., 2017; Cho et al., 2020), while sodium adducts prevail in MALDI‐MS (Zhong et al. 2018). Permethylation together with liquid‐liquid extraction or reversed phase (RP)‐solid phase extraction (SPE) represents an efficient clean‐up step for MS analysis of glycans, corresponding workflows are well established and have been adapted to the 96‐well plate format (Shajahan et al., 2019; Shubhakar et al., 2016). It should be noted that the usage of liquid‐liquid extraction (as purification method) results in a loss of hydrophilic analytes, such as sulphated *O*‐glycans. The full benefit of the very high sensitivity of the MS detection of permethylated glycans is not realized in biomedical and biopharma applications, as the sample preparation still requires considerable amounts of glycans and glycoconjugates. Hence, innovation and particularly miniaturization of permethylation sample preparation is seen as a key point for realizing ultrahigh sensitivity workflows. A recent publication on the use of carbon nanoparticles for an SPE cleanup of permethylated glycans before MALDI‐MS provides a promising perspective (Zhong et al. 2018). However, it should be noted that permethylation also comes with its downsides such as rather time‐consuming protocols, the usage of toxic reagents and, as with most derivatization procedures, potential by‐products that increase sample heterogeneity.

Over a decade ago, it was demonstrated by Naven and Harvey that a ten‐fold increase in MALDI‐MS sensitivity could be obtained compared to underivatized glycan detection when a cationic reducing‐end label was introduced through hydrazone formation (Naven & Harvey, 1996). One of the major advantages of this procedure is that no additional clean‐up procedures are required as, during the reaction, no salts are used or produced, allowing immediate analysis of the sample. An overview is provided of hydrazone‐based reducing end labeling procedures for MS analysis by Lattová and Perreault (Lattová & Perreault, 2013). Next to derivatizing the reducing end of the glycan, recent studies focused on the stabilization of the sialylated species. This allows the distinction between differently linked sialic acids, next to an improved detection level in positive ionization mode (de Haan et al., 2020; Pongracz et al., 2019).

### 3.5 Glycoproteomics

To gain a better understanding of the fundamental role of glycans and glycoconjugates, glycoprotein‐based approaches are required, providing insights in the macro‐ and microheterogeneity of protein glycosylation as well as integrating the glycomics and proteomics fields. For this purpose, general bottom‐up proteomics workflows are often applied by performing a proteolytic digest. In cases where a specific nonabundant protein is targeted from a complex biological sample, the sensitivity can be increased by introducing a depletion or immunoaffinity step (e.g., SDS‐PAGE or immunoaffinity) for removing the most abundant proteins or to selectively capture the protein of interest, respectively (Ruhaak et al., 2018). Additional glycopeptide enrichment strategies, such as HILIC‐SPE, are often applied as glycopeptides often occur in substoichiometric quantities due to glycosylation microheterogeneity and tend to ionize less efficiently compared to most other peptides (Stavenhagen et al., 2013). Due to the various advancements that have been made regarding sample preparation procedures as well as technical improvements the glycoproteomics field is rapidly evolving (Riley et al., 2021; Thomas & Scott, 2021).

### 3.6 Clean‐up steps

#### 3.6.1 Porous graphitized carbon solid‐phase extraction (PGC‐SPE)

PGC‐SPE is regularly used for glycan clean‐up, and often facilitates the separation between neutral and acidic glycans (Packer et al., 1998; Stavenhagen et al., 2015). Next to ready‐to‐use PGC cartridges, miniaturized PGC‐SPE devices are applied in high sensitivity glycomics, including self‐packed micro‐SPE devices in, for example, pipette tips with filters (Delafield & Li, 2020; Xin et al., 2012). Commercial Hypercarb™ PGC SPE 96‐well plates (Thermo Fisher Scientific) come in different sizes ranging from 10 to 100 mg bed weight, but miniaturized versions are self‐prepared on 96‐well plate filter plates using bulk PGC material (Zhang, Madunić, et al. 2020). While monosaccharides often show insufficient retention for PGC purification, disaccharides tend to be at least partially retrieved by PGC‐SPE, most common glycomic PGC‐SPE applications target glycans as opposed to mono‐ and disaccharides. Large multiantennary glycans with multiple LacNAc repeats, sialic acids as well as other negative charges (in particular glycopeptides) can be hard to elute from PGC and hence may be poorly covered in workflows including PGC‐SPE (Stavenhagen et al., 2015). Elution of glycans from PGC is generally achieved in an MS‐compatible manner, with acetonitrile‐water mixtures which optionally contain a volatile acid (formic acid or trifluoroacetic acid) to facilitate the elution of acidic glycans (Packer et al., 1998).

#### 3.6.2 Hydrophilic interaction liquid chromatography

HILIC is highly suitable for glycan and glycoconjugate purification, due to the hydrophilic character of the glycan moiety, which can be applied as an enrichment step before analysis or as a chromatography mode for LC (see Section 4.2). A range of different stationary phases is available including amine‐based stationary phases which show under low ionic strength conditions mixed‐mode retention with a contribution of ionic interactions (Wuhrer et al., 2009). HILIC stationary phases that do not retain, based on ionic interactions, are, for example, amide‐ and diol‐functionalized beads, polyacrylamide‐based beads as well as cellulose‐based materials such as microcrystalline cellulose and cotton (Wuhrer et al., 2009). Microcrystalline cellulose can be packed as a slurry in columns of different sizes and can, for example, be packed in 96‐well plates for miniaturized SPE. Cotton can be used in the form of cotton wool (Selman et al., 2011) or cotton thread (Vreeker et al., 2018). The latter one is particularly suitable for preparing micro‐SPE devices in pipetting tips on a robotics platform in high‐throughput mode (Vreeker et al., 2018). Overall, HILIC‐SPE is often applied for the purification of glycans, glycan alditols, many different reducing end‐tagged glycans, and glycopeptides. While the aglycons are modulating HILIC retention, an efficient HILIC enrichment can often be achieved by minor adjustments of the loading and washing conditions, for example, change of organic solvent or concentration (Mysling et al., 2010). Due to the hydrophilic environment, it might be challenging to enrich via HILIC when large, hydrophobic aglycons are present—be it fluorescence tags or peptide portions—as they may results in solubility issues during loading, washing and elution steps. It should be noted that for glycoproteomic applications hydrophilic non‐glycosylated peptides can be coenriched (Mysling et al., 2010).

#### 3.6.3 Reverse phase solid‐phase extraction (RP‐SPE)

RP‐SPE can be used for clean‐up after reducing end‐labeling (Ruhaak et al., 2010) or for purification of glycoconjugates such as glycolipids and glycopeptides. In glycolipid analysis, the enzymatic release of the glycan head group is often followed by a RP‐SPE step for removing ceramides and detergents, retrieving the released glycans in the flow‐through (Jongsma et al., 2021). RP‐SPE is applicable in a broad range of dimensions, and there are various miniaturized formats available such as ZipTip® Pipette Tips (Merck Millipore) which are particularly suitable for sample cleanup before MS analysis.

## 4 MS GLYCOMICS: INTEGRATED WORKFLOWS

### 4.1 Capillary electrophoresis mass spectrometry

**Figure 4:**
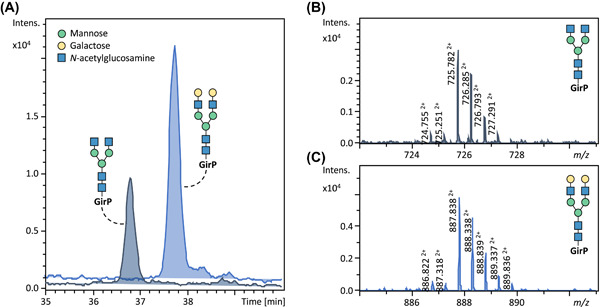
Sensitivity assessment of sheathless CE‐ESI‐MS analysis of glycans using DEN gas. Glycans were subjected to a glycomic workflow including derivatization (dimethylamidation) and reducing‐end labeling with GirP using a starting amount of 5 femtomole. (A) Extracted ion electropherograms of the glycan standards (H3N4 and H5N4) with a consumed amount of 20 attomole. Spectra of the doubly charged analytes corresponding to H3N4 and H5N4are illustrated in (B) and (C), respectively. GirP illustrates the label attached to the glycans. Figure adapted with permission from Lageveen‐Kammeijer et al. (2019). Copyright © 2019. DEN, dopant‐enriched nitrogen; GirP, Girard's reagent P [Color figure can be viewed at wileyonlinelibrary.com]

CE‐MS is a powerful technique when it comes down to sensitivity. Just recently a study reported on the sheathless‐based CE‐MS detection of sialic acid‐derivatized *N*‐glycans labeled with Girard's reagent P (GirP) (Lageveen‐Kammeijer et al., 2019). The workflow relies on an established, single pot sialic acid derivatization chemistry that allows to differentiate sialic acid linkages and is a further development of a protocol originally described by Wheeler et al. (2009). Sialic acid derivatization is followed by a simple, miniaturized cotton HILIC SPE step performed using a cotton thread of several 100 microgram as stationary phase, with elution of the derivatized glycans in 10 microliter of water (Reiding et al., 2016). An aliquot of the eluate is then used for single‐step GirP derivatization followed by CE‐MS analysis. For CE‐MS, the use of a sheathless porous capillary sprayer comes with excellent sensitivity and reasonable robustness for glycan analysis. An important aspect in boosting sensitivity is the use of dopant‐enriched nitrogen (DEN) gas to promote ionization of labeled glycans. While this approach has previously been established for the analysis of glycopeptides (Kammeijer et al., 2016), it is similarly powerful for the ESI analysis of glycans. For CE‐MS, a hardware adaptation was induced in the form of a tube concentrically surrounding the spray tip for directing the DEN gas flow. The sensitivity gain by implementing DEN gas is approximately three‐fold for *N*‐glycans (Lageveen‐Kammeijer et al., 2019) and 25‐fold for model glycopeptides (Kammeijer et al., 2016). Overall, the method shows an excellent sensitivity of the final CE‐MS glycan detection step, with low attomole amounts of glycans being detected (Figure 4 and Table 1), mainly due to the miniaturized ESI setup featuring favorable ionization conditions. For the overall workflow, likewise, a very high sensitivity is achieved, with five femtomole of a protein‐linked glycan species present in the glycoprotein starting material allowing confident MS detection and relative quantification (Lageveen‐Kammeijer et al., 2019). Another recent publication, by the Ivanov group, demonstrated that the implementation of this DEN gas was also beneficial for the analysis of APTS‐labeled glycans measured in negative mode (Marie et al., 2021). For this purpose, isopropanol was selected as the most suitable dopant, resulting in an approximately 100‐fold increase in sensitivity, allowing the detection of glycans in extracellular vesicles from <500 nanoliter of blood (injected amount) and reporting on the identification of more than 400 *N*‐glycan structures.

**Figure 5:**
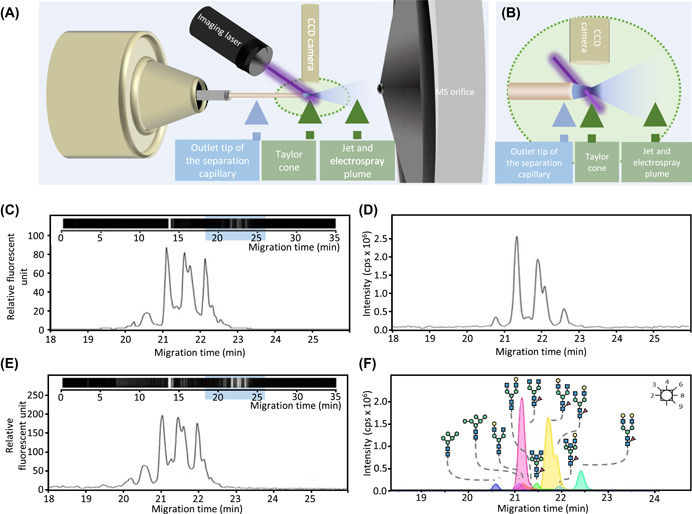
CE‐MS separation of APTS labeled IgG1 N‐glycans hyphenated with fluorescence detection. A schematic representation of the setup for iLIF detection at the Taylor cone of CE‐ESI‐MS is provided in (A), and a magnification of the situation at the Taylor cone is given in (B). (C) and (E) provide the fluorescence signal obtained at the capillary tip and in the Taylor cone, respectively. The inserts indicate the observed fluorescent signal and blue highlights the electropherogram region. (D) and (F) illustrate the obtained MS signal in negative ionization mode. Extracted ion electropherograms of the most abundant *N*‐glycans in human IgG1 are depicted in (F). Figure adapted with permission from (Szarka et al., 2019). Copyright © 2019, American Chemical Society. APTS, 9‐aminopyrene‐1,4,6‐trisulfonic acid; iLIF, imaging laser‐induced fluorescence [Color figure can be viewed at wileyonlinelibrary.com]

Recently, the CE‐MS detection of APTS‐labeled glycans has been improved by allowing high sensitivity fluorescence detection in the Taylor cone (Figure 5) (Szarka et al., 2019). This very promising innovation improves the sensitivity using the fluorescence detection at the Taylor cone (Figure 5E) compared to the detection in capillary‐scale flow chambers (Figure 5C). One may assume that the fluorescence detection in the Taylor plume would likewise be an elegant solution for the combination with MS detection in nanoLC mode.

### 4.2 HILIC‐ and RP‐LC‐MS of tagged glycans

The LC‐MS detection of fluorescently labeled glycans likewise showed a major push towards high sensitivity detection. Next to CE‐LIF, HILIC‐UPLC‐FLD is one of the most applied separation principles for detection of fluorescently labeled glycans (Reusch et al., 2015). An important aspect determining sensitivity is the choice of the tag—both for fluorescence and MS detection (Pabst et al., 2009).

In terms of coupling chemistry, the most common tags used for HILIC fall into two groups: conventional tags such as 2‐aminobenzoic acid (2‐AA), 2‐AB, APTS and, more recently, tags through reductive amination such as procainamide (Kozak et al., 2015; Ruhaak et al., 2010). Of those, APTS shows very high fluorescence sensitivity (Pabst et al., 2009), yet its use for high sensitivity applications can be compromised by sometimes only mediocre labeling yields. While APTS is mostly used for CE applications, it is also very much suitable for HILIC with FLD. For MS detection APTS is a very challenging label, and despite its highly acidic nature ESI‐MS detection of tagged glycans is generally performed in positive‐ionization mode (Bunz, Cutillo, et al., 2013; Bunz, Rapp, et al., 2013). 2‐AA and 2‐AB are both performing similarly well in positive‐ion mode MS detection, with 2‐AA likewise allowing sensitive negative‐mode detection in both MALDI‐ and ESI‐MS applications (Anumula & Dhume, 1998; Ruhaak et al., 2008; Reiding et al., 2017).

An alternative group of tags that are specifically used for *N*‐glycan analysis are the amine‐reactive reagents which are targeting the glycosyl amine that is generated by PNGase F‐mediated release of *N*‐glycans (Lim et al., 2019). These tags (e.g., RFMS and procainamide; Figure 3) often combine fast, facile workflows with high sensitivity in fluorescence and MS detection (Keser et al., 2018). The sensitivity gain, relative to the gold standard 2‐AB tag, has been reported to be approximately four‐fold in FLD and more than 50‐fold in MS detection (Keser et al., 2018). Although these gains may strongly depend on the chosen analytical platforms and settings. These tags are largely used in biopharma applications, where the high sensitivity is not per se needed, as sample amounts are hardly ever limiting, and the fast, mild, and facile sample preparation might be more important. Nonetheless, these tags may also have major potential for biomedical research applications.

While the solvent mixtures used for *N*‐glycan elution are often favorable for online ESI‐MS detection (low concentrations of volatile acid or salt, high concentrations of acetonitrile), the column dimensions and flow conditions prohibit to operate at its highest‐sensitivity for MS detection: oftentimes, analytical‐scale or microbore separations are performed, and miniaturization of HILIC‐FLD‐MS or HILIC‐MS of *N*‐glycans to the capillary‐scale or nano‐scale is seldomly achieved (Wuhrer et al., 2004, 2004); this may be due to a lack of commercially available columns, as well as the often limited robustness and separation performance of these setups.

When pushing HILIC‐FLD or HILIC‐MS detection of reducing end‐labeled glycans towards high sensitivity, miniaturization, and reduction of the complexity of the workflow become important factors. An established, miniaturized and multiplexed workflow for the analysis of 2‐AA‐labeled glycans relies on a single HILIC‐SPE cleanup step (Ruhaak et al., 2008). By moving to nanoRP‐LC‐MS as a higher‐sensitivity detection method, this workflow becomes suitable for the analysis of minute sample amounts, as discussed in a recent review on RP‐separation methods for glycan analysis (Vreeker & Wuhrer, 2017). In RP‐LC‐MS, elution conditions are often favorable for MS detection for late‐eluting analytes, while early eluting peaks may show somewhat lower sensitivity in MS detection due to low organic modifier content in the eluent. The analysis of 2‐AA‐labeled glycans by nanoRP‐LC‐MS separation with a qTOF‐MS detection system employed with DEN gas featured an approximately 100 times higher sensitivity than HILIC‐FLD using a 2 mm column ID setup, with the sensitivity of the nanoRP‐LC‐MS setup being in the attomole range (Table 1), while FLD showed low femtomole sensitivity (unpublished results).

### 4.3 Porous graphitized carbon liquid chromatography mass spectrometry (PGC‐LC‐MS)

A particularly versatile approach for the in‐depth structural characterization of a broad range of glycans is PGC‐LC‐MS and has been pioneered by Packer et al. (1998). Compared to other platforms, PGC‐LC‐MS has several advantageous features: the PGC stationary phase shows outstanding glycan isomer separation, particularly in the case of glycans and glycoconjugates with only a small aglycone (Ruhaak et al., 2009). Often anomer separation is observed for reducing‐end glycans, resulting in rather complex chromatograms (Xu et al., 2020). The complexity can be reduced by analyzing glycan alditols (obtained after reduction) as they feature migration patterns that are not further influenced by anomer separation (Jensen et al., 2012; Zhang, Madunić, et al., 2020). Therefore, PGC‐LC‐MS is often used for the analysis of glycan alditols. PGC‐LC‐MS shows extensive isomer separation for *N*‐glycans, *O*‐glycans and glycosphingolipid‐derived glycans (Anugraham et al., 2015; Jensen et al., 2012; Zhang, van Die, et al., 2020; Zhang, Madunić, et al., 2020), thereby, considerably enhancing the MS structural elucidation of glycans. PGC‐LC‐MS is a particularly good match with the analysis of *O*‐glycan alditols (product of reductive beta‐elimination), due to its excellent separation power of isomeric species resulting in obtaining glycan profiles with high structural diversity (Madunic et al., 2021). For *N*‐glycans and glycolipid glycan moieties, the reduction step under alkaline conditions comes with a downside, that is, the loss of alkaline‐labile modifications such as *O*‐acetyl groups. These losses are avoided by reducing the glycans before analysis. The most applied ionization mode for PGC‐LC‐MS is negative ion mode, due to informative cross‐ring cleavages of glycan alditols in MS/MS. In addition, negative‐mode collision induced dissociation experiments, as opposed to positive‐ion mode, show a pronounced stability of, for example, terminal fucose residues (Harvey, 2005; Wuhrer & Deelder, 2005).

**Figure 6:**
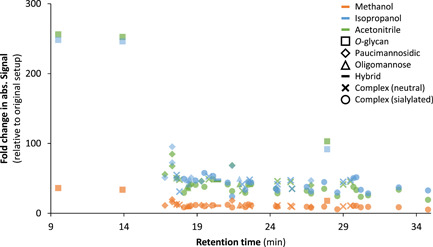
Effect of different post‐column makeup flow supplements on *N*‐ and *O*‐glycan detection. The fold change of the AUC relative to the original setup is plotted with supplements consisting of methanol (orange), isopropanol (blue) or acetonitrile (green), resulting in a net concentration of 57% organic solvent (*v/v*) at the ion source. *N*‐ and *O*‐glycans were measured separately. Each symbol indicates a specific glycan subtype. Figure reused from (Hinneburg et al., 2019). AUC, area under the curve [Color figure can be viewed at wileyonlinelibrary.com]

To further improve the detection sensitivity of a PGC‐LC‐MS platform, a recent study investigated the usage of a post‐column make‐up flow (PCMF) using various organics (Hinneburg et al., 2019). For this purpose post‐column addition of methanol, isopropanol and acetonitrile was compared to a conventional PGC‐LC‐MS setup (Jensen et al., 2012). Overall, a 30‐ up to a 100‐fold increase in glycan signal intensity was obtained for various released *N*‐glycans with an even up to 250‐fold increase for *O*‐glycans (Figure 6). As expected, this resulted in higher‐quality MS/MS spectra. While all glycans benefited from the PCMF, the early eluting glycans are the main beneficiaries, showing the highest enhancement in intensity and area under the curve. The best sensitivities were obtained by adding 100% acetonitrile or isopropanol, which resulted in a 57% (*v/v*) net concentration at the ionization source.

Building on these results, another study investigated whether applying DEN gas in the electrospray ionization source could be used to increase detection sensitivity for glycan alditols in negative‐ion mode (Madunic et al., 2021). Here, similar findings were obtained compared to the PCMF platform, resulting in an increased sensitivity especially for the early‐eluting glycans, where the mobile phase contained very low concentrations of organics. While isopropanol as a dopant provided the best result regarding fragmentation spectra, methanol seemed to be more suitable for improved signal‐to‐noise ratios. Overall, it was estimated that glycans could be detected at mid femtomole amount (Table 1).

Sample preparation workflows for PGC are compatible with high sensitivity analysis of *N*‐glycans and *O*‐glycans from purified proteins, but also from tissue samples (Hinneburg et al., 2017). Recently, the sample preparation workflow for nanoPGC‐LC‐MS was transferred to 96‐well plate format, without compromising on precision and sensitivity (Zhang, Madunić, et al. 2020). High sensitivity applications of nanoPGC‐LC‐MS include the analysis of *N*‐ and *O*‐glycomic signatures from tumor tissue regions of approximately 2,000 cells prepared by laser capture microdissection (Hinneburg et al., 2017). However, nanoPGC‐LC‐MS is only used by a small number of laboratories, as these column formats are not commercially available, and columns are often packed in‐house (Karlsson et al., 2004). Capillary‐scale and microbore PGC‐LC‐MS is more common, yet with lower sensitivity.

### 4.4 Ion mobility spectrometry MS (IMS‐MS)

An emerging separation technique in the glycomics field is IMS coupled to MS, due to its ability to separate isomeric species in the gas‐phase. Recently a comprehensive overview of advancements of IMS‐MS for glycomic applications has been published by Chen et al. (2018). Like CE, the analytes are separated by creating an electric field based upon their mobility, which is influenced by the hydrodynamic volume and charge of an analyte. However, as opposed to CE, the analytes are ionized and interact with a carrier gas. The powerful technique has already been demonstrated for its ability to separate isomeric glycan species (Pallister et al., 2020; Sastre Toraño et al., 2021) or to detect variations in site‐occupancy (Guttman & Lee, 2016). While IMS does not directly improve the sensitivity of the analysis, it does provide valuable structural information compared to the conventional separation platforms such as CE or LC. However, it should be noted that an improved peak capacity and separation is observed when IMS is combined with one of these separation platforms (allowing a two‐dimensional separation) (Lareau et al., 2015).

### 4.5 MALDI MSI

**Figure 7:**
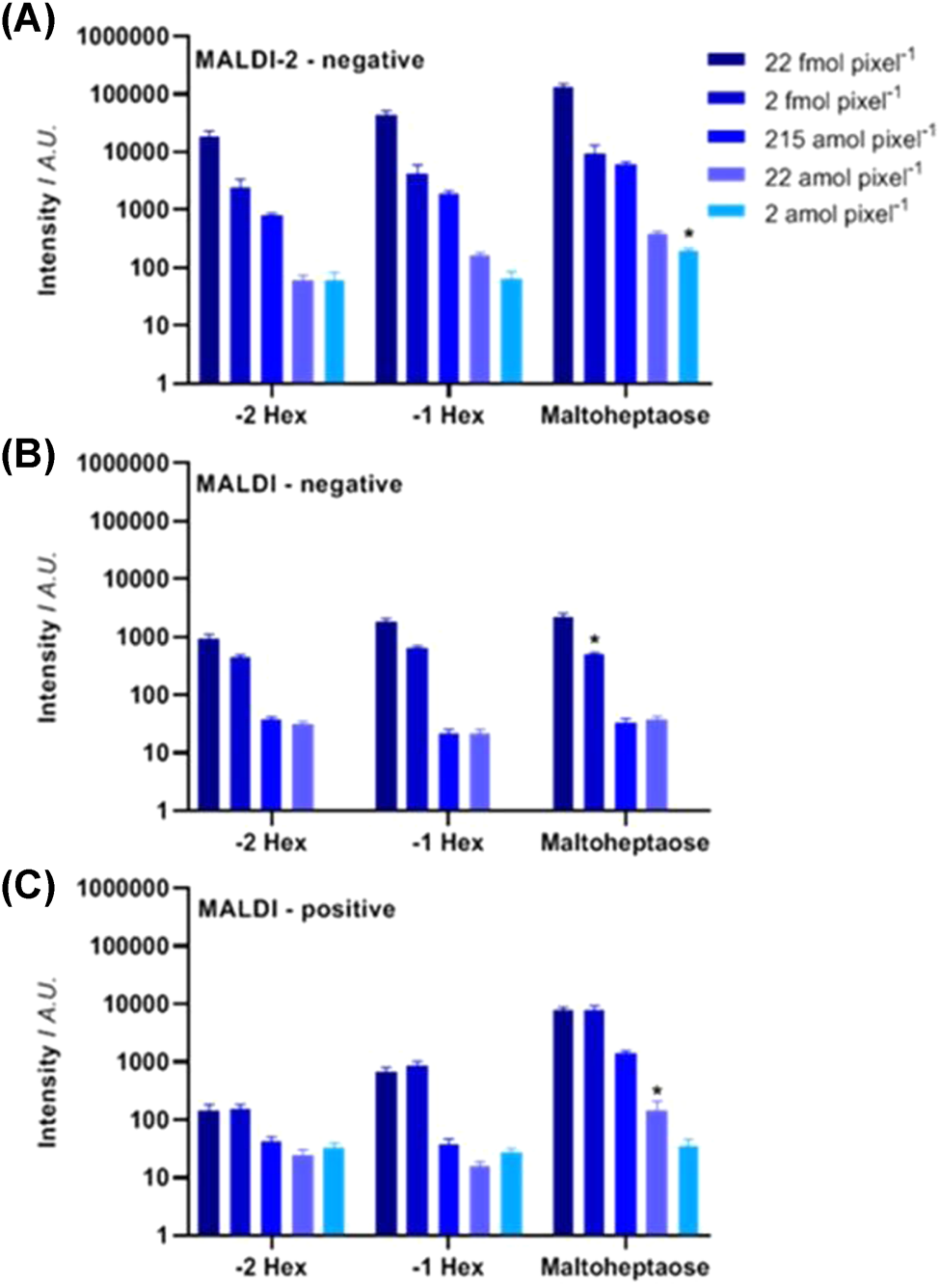
Sensitivity gain by MALDI‐2 of glycans in negative‐ion mode. A dilution series of maltoheptaose was spray‐coated on glass slide and analyzed by (A) MALDI‐ 2‐MS in negative ion mode, (B) MALDI‐MS in negative ion mode, and (C) MALDI‐MS in positive ion mode. The bar graphs show the mean intensity of the performed experiments (*n* = 5). The lower limit of detection (signal‐to‐noise ratio ≥3) is indicated with an *. Error bars represent the standard error of the mean. Figure adapted with permission from (Heijs, Potthoff, et al., 2020). Copyright © 2020, American Chemical Society. MALDI‐MS, matrix‐assisted laser desorption/ionization mass spectrometry [Color figure can be viewed at wileyonlinelibrary.com]

MSI of *N*‐glycans is generally performed in MALDI mode and has been pioneered by Drake and co‐workers (Powers et al., 2013). A pixel of an *N*‐glycan MSI experiment generally covers a single or a few cells, dependent on the tissue type and the chosen experimental conditions. Hence, single cell *N*‐glycomic analyses by MALDI‐MSI are within reach. In addition, the information content can be increased by applying on‐tissue derivatization of glycans by linkage‐specific sialic acid derivatization, thereby allowing facile differentiation of linkage isomers based on the linkage‐specific mass shifts (Holst et al., 2016). Recent applications of *N*‐glycan MSI are largely focused on tumor tissue analysis (Boyaval et al., 2020; Carter et al., 2020; Drake et al., 2017, 2020; Heijs, Holst‐Bernal, et al., 2020; McDowell et al., 2020). A variation of the *N*‐glycan MSI approach has recently been established for antibody array‐based glycoprotein enrichment from biofluids with subsequent *N*‐glycan imaging (Black et al., 2019). By combining laser‐induced post ionization with conventional MALDI‐MSI in negative mode, or so called MALDI‐2, the detection sensitivity could be increased for both positive ionization mode [M + Na]^+^ as well as negative ionization mode [M‐H]^‐^ by one and three orders of magnitude, respectively (Heijs, Potthoff, et al., 2020). This resulted in a low limit of detection of two attomole per pixel in negative‐ion mode for MALDI‐2 compared to two femtomole per pixel in negative ion mode and 22 attomole per pixel in positive ion mode for conventional MALDI‐MSI (Figure 7 and Table 1). Of note, preliminary results revealed that MALDI‐2 was only advantageous in negative mode as MALDI‐2 does not seem to outperform conventional MALDI‐MSI in positive mode when salt‐adducts are formed.

### 4.6 Glycopeptide analysis

Glycopeptide analysis is a very attractive approach for high sensitivity glycomic analysis and sample preparation workflows are often very similar to those of high sensitivity quantitative proteomics. Due to the often substoichiometric nature of protein glycosylation and the often‐poor ionization properties of glycosylated peptides, sensitivity of glycopeptide‐based glycoproteomics tends to severely lag that of other mainstream bottom‐up proteomics applications. Nevertheless, due to the nanoLC setup (which is commonly used in proteomics) glycopeptide analyses in nanoRP‐LC‐MS mode tend to show good sensitivities. An important factor is the choice of the ionization conditions: the use of DEN gas—in particular acetonitrile‐enriched nitrogen gas—was reported to result in a tremendous boost of signal intensities (Alagesan & Kolarich, 2019) and has found its way into routine applications (Falck et al., 2017; Larsen et al., 2021).

Importantly, nanoRP‐LC‐MS analysis of glycopeptides can be embedded into simple, miniaturized workflows for high sensitivity site‐specific glycosylation analysis as exemplified for the Fc glycosylation analysis of antibodies. While bulk IgG analysis from serum or plasma does not generally require high sensitivity approaches due to the high concentration of IgGs in these biofluids (Falck et al., 2017), there are multiple applications that come with only minute amounts of IgG and hence require high sensitivity. This include the analysis of IgG glycosylation from other biofluids such as saliva (Plomp et al., 2018), the analysis of antigen‐specific IgG from different biofluids (Larsen et al., 2021; Scherer et al., 2009), and the analysis of IgG Fc glycosylation from small‐scale or B‐cell in vitro cultures (Wang et al., 2011). For more information on glycopeptide analysis using MS, we refer to various excellent in‐depth reviews (Cao et al., 2016; Chernykh et al., 2021; Dalpathado & Desaire, 2008; Ruhaak et al., 2018).

### 4.7 Intact glycoprotein analysis

**Figure 8:**
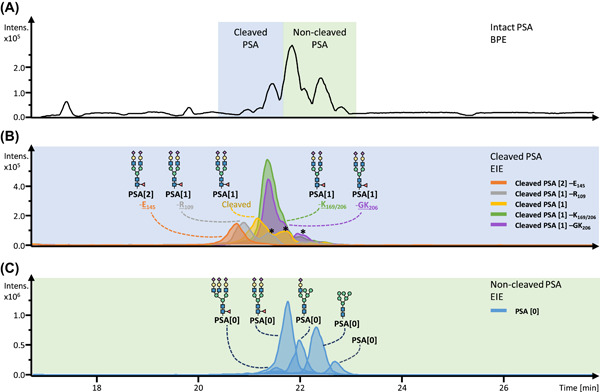
Intact analysis using CE‐ESI‐MS of PSA captured from a patient pool. (A) BPE of intact urinary PSA. (B) EIEs reveal various PSA proteoforms with and without internal cleavages. The proteoforms are illustrated with their most abundant glycan. The square brackets indicated the number of internal cleavages, followed by a potential loss of amino acid(s). (C) EIEs of the most abundant glycan structures (tri‐, di‐, mono‐sialylated and high mannose type) as well as the nonglycosylated proteoforms without an internal cleavage. Overlapping *m*/*z* values are indicated with an *. Figure adapted with permission from (Moran et al., 2021). Copyright © 1969, Elsevier. BPE, base peak electropherogram; EIE, extracted ion electropherogram; PSA, prostate‐specific antigen [Color figure can be viewed at wileyonlinelibrary.com]

Intact glycoprotein analysis has a vast potential for sensitive glycosylation analysis, due to the mostly simple and straight‐forward sample preparation workflows. Recently, mass spectrometers and corresponding methods have been tailored for the sensitive analysis of intact (glyco)proteins, be it under native or denatured conditions. Sample preparation for intact glycoprotein analysis is often particularly simple, including, for example, only a buffer exchange or an affinity capturing step (Donnelly et al., 2019; Lacey et al., 2001). MS analysis can then be achieved by direct infusion ESI‐MS, or by online ESI‐MS in RP‐LC‐MS or CE‐MS configuration (Donnelly et al., 2019; Gstöttner et al., 2020). It should be noted that these workflows are particularly well applicable for the analysis of biopharmaceuticals or glycoproteins that are present at high concentrations in biofluids. However, in these cases, high sensitivity is often not essential as high amounts of the glycoprotein are generally available. In contrast, high sensitivity is of relevance for complex mixtures such as plasma or serum, especially when the targets are glycoproteins that are present at low concentrations. For example, a recent publication investigated the various proteoform variants of prostate‐specific antigen (PSA) present in urine of prostate cancer patients (Moran et al., 2021). Next to PSA glycoforms, six proteolytic cleavage variants could be identified on an intact level (Figure 8). While further research is still required, the intact analysis of PSA in combination with a bottom‐up approach provides an important basis for future studies that focus on biomarker characterization in the field of prostate cancer. Especially in assays like these, high‐efficiency sample preparation is important, for example, via affinity capturing. Due to its overall simplicity, intact glycoprotein analysis has vast potential for high sensitivity applications, and we expect this field to evolve quickly in the coming years.

### 4.8 Middle‐up and middle‐down glycoprotein analysis

**Figure 9:**
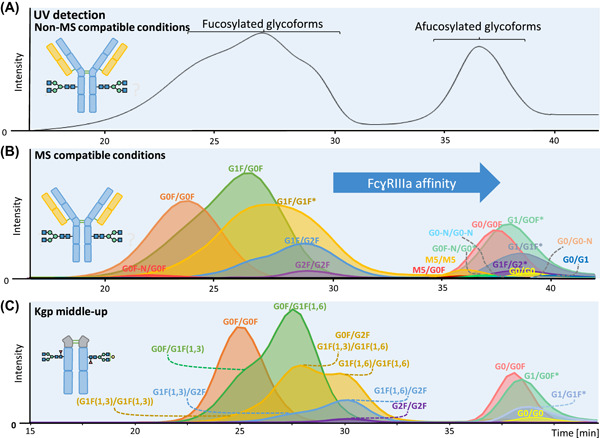
FcɣRIIIa AC of a therapeutic mAb. (A) UV chromatogram reported with non‐MS compatible conditions. AC‐MS under MS‐compatible conditions represented by extracted ion chromatograms of detected glycoforms on intact level (B) or Kgp middle‐up level (C). The * indicates multiple possibilities, the most probable glycoform is presented. Figure adapted from Lippold, Nicolardi, Domínguez‐Vega, et al. (2019) and Lippold, Nicolardi, Wuhrer, et al. (2019). Copyright © 2019. Published with license by Taylor & Francis Group, LLC. AC, affinity chromatography [Color figure can be viewed at wileyonlinelibrary.com]

Intact characterization of biopharmaceuticals can be complemented by the mass analysis of individual subunits which is known as the middle‐up approach (Lermyte et al., 2019). In case these individual subunits are characterized by MS/MS the approach is referred to as middle‐down. For example, proteolytic cleavage of the hinge region of antibodies is well‐established to yield an Fc‐ and a Fab‐domain and is often performed in combination with reduction of disulfide bridges. The main advantage over direct intact analysis is a reduced molecular weight of individual analytes and the assignment of specific modifications to individual subunits. For middle‐up and middle‐down MS analysis of antibodies various separation platforms are available such as size exclusion chromatography (Haberger et al., 2016), RPLC (Wang et al., 2018), HILIC (D'Atri et al., 2017; Sénard et al., 2020) or CE (Gstöttner et al., 2020; Sénard et al., 2020). Especially in combination with affinity chromatography a powerful platform is being created, providing insights how individual glycoforms on an antibody interact with an immobilized receptor. For this procedure high sensitivity is desired, to detect and characterize low abundant glycoforms which might play a crucial role in, for example, antibody‐dependent cell‐mediated cytotoxicity (Figure 9) (Lippold, Nicolardi, Domínguez‐Vega, et al., 2019; Lippold, Nicolardi, Wuhrer, et al., 2019).

In this review we only highlighted a few novel applications and developments in the field of glycopeptide, intact glycoprotein as well as middle‐up and middle‐down analysis and we would like to refer the reader to other reviews for a more extensive overview regarding recent advancements made on these topics (Camperi et al., 2020; Narimatsu et al., 2018; O'Flaherty et al., 2018; Ohyama et al., 2020; Ruhaak et al., 2018; Xiao et al., 2018, 2019; Yang, Franc, et al., 2017; Yu et al. 2018).

## 5 CONSIDERATIONS

Here a bird's eye view was provided of various MS based techniques and their corresponding sensitivities (Table 1). While most glycomic studies allege to have developed a sensitive method, only few studies actually assess the limit of detection (e.g., by using internal standards). To enable a better comparison between the significance and complimentarity of the various aplications, there is an urgent need for studies that support their sensitivity claims by properly designed experiments. However, the heterogeneous character of the glycoconjugates as well as the lack of standards could be considered the main bottleneck for this assessment. Due to recent progress in chemoenzymatic synthesis, the preparation of structurally defined, stable isotope‐labeled oligosaccharide and glycoconjugate standards has become feasible (Etxebarria & Reichardt, 2016; Guberman & Seeberger, 2019; Liu et al., 2019).

Moreover, there seems to be no general consensus in the glycomics field as what is considered the true sensitivity of an assay. While an absolute sensivitity for determining a single glycan can be given, similar to other omics fields, it may be debatable whether this is the most relevant information, as a single glycan does not provide direct insights in the biological pathway or what alterations might occur during a disease. Therefore, the scope should be clearly defined before the study, for example, the top 10 most abundant glycans should be characterized which may often result in a 100‐fold higher limit of detection as well as sample consumption compared to the detection limit for merely the most abundant glycan. Additionally, while it is important to know the sensitivity of the analtyical platform, it is more critical and informative when the required starting amount is defined as often more sample is needed to perform the entire analytical procedure compared to what is being needed or consumed for the final detection step. Eventually the glycomic field will be evolving to single cell analysis, however, this will only be informative when more than a single glycan species can be detected.

This review mainly focused on the analysis of mammalian *N*‐ and GalNAc‐type *O*‐linked glycans and, while not discussed here, we would like to highlight that the other glycan classes are equally significant (Puri et al., 2020; Schjoldager et al., 2020; Zhang et al., 2019; Zhang, van Die, et al., 2020). However, due to the lack of characterization tools and generic protocols, these analytes are still underexplored.

To gain a better understanding of the disease progression as well as to unravel the biology behind the alterations it will be essential to not only pinpoint changes in a glycomic profile of various diseases but also to integrate this information with other omics fields (Kellman & Lewis, 2021). The first steps are already initiated by integrating glycomic and glycoproteomic data with previously published datasets (e.g., transcriptomics) (Madunic et al., 2021) as well as to correlate it with data on The Human Tissue and Blood Atlas (part of the Human Protein Atlas) (Kawahara et al., 2021) While these studies reused publised data by other research groups, actually the same patient or cell (line) should be explored with all different omics applications, to minimize confounding by batch‐to‐batch and interindividual variation. Bringing the various omics disciplines and expertise together provides valuable insights on how the glycome and glycoproteome are regulated via glycosidases and glycosyltransferases (Blazev et al., 2021).

## 6 CONCLUSIONS

In this review we presented recent developments in the field of high sensitivity MS based glycomics. This emerging field still lags analytical sensitivity when compared to the genomics and transcriptomics field and to a lesser extent proteomics and metabolomics. Though, recent developments towards higher sensitivity glycomics are encouraging, and detection of glycan signatures at single molecule or single cell level have come within reach. To gain a better understanding of underlying molecular mechanisms it is important to not solely focus on glycomics signatures but rather incorporate these with omics applications. Overall, high sensitivity MS glycomics will often require a reduction in number and complexity of sample preparation steps, and a balance between glycan structural information versus glycome coverage and sensitivity. Glycopeptide‐centered approaches increasingly complement glycomic approaches and perform very well regarding sensitivity as they build on established as well as quickly evolving high sensitivity proteomic workflows. Moreover, intact glycoprotein analysis by MS has a huge potential for high sensitivity glycomic analyses, with relatively straight‐forward sample preparation as an advantage, and we expect that high sensitivity intact protein analysis will claim its place in the field.
